# Respiratory Syncytial Virus (RSV) G Protein Vaccines With Central Conserved Domain Mutations Induce CX3C-CX3CR1 Blocking Antibodies

**DOI:** 10.3390/v13020352

**Published:** 2021-02-23

**Authors:** Harrison C. Bergeron, Jackelyn Murray, Ana M. Nuñez Castrejon, Rebecca M. DuBois, Ralph A. Tripp

**Affiliations:** 1 Department of Infectious Diseases, University of Georgia, Athens, GA 30605, USA; harrison.bergeron@uga.edu (H.C.B.); jcrab@uga.edu (J.M.); 2 Department of Biomolecular Engineering, University of California-Santa Cruz, Santa Cruz, CA 95064, USA; amnunezc@ucsc.edu (A.M.N.C.); rmdubois@ucsc.edu (R.M.D.)

**Keywords:** respiratory syncytial virus, RSV, vaccine, G protein, G glycoprotein, antibodies, CX3CR1

## Abstract

Respiratory syncytial virus (RSV) infection can cause bronchiolitis, pneumonia, morbidity, and some mortality, primarily in infants and the elderly, for which no vaccine is available. The RSV attachment (G) protein contains a central conserved domain (CCD) with a CX3C motif implicated in the induction of protective antibodies, thus vaccine candidates containing the G protein are of interest. This study determined if mutations in the G protein CCD would mediate immunogenicity while inducing G protein CX3C-CX3CR1 blocking antibodies. BALB/c mice were vaccinated with structurally-guided, rationally designed G proteins with CCD mutations. The results show that these G protein immunogens induce a substantial anti-G protein antibody response, and using serum IgG from the vaccinated mice, these antibodies are capable of blocking the RSV G protein CX3C-CX3CR1 binding while not interfering with CX3CL1, fractalkine.

## 1. Introduction

Respiratory syncytial virus (RSV) is an important human respiratory tract pathogen of infants and the elderly causing pneumonia and bronchitis [1,2,3]. RSV is a primary cause of hospitalization for respiratory tract illness in young children, and may cause serious lower respiratory tract disease in 20–30% of children, requiring hospitalization [4]. RSV is a non-segmented negative-sense RNA virus containing two nonstructural (NS1 and NS2) genes followed by nucleocapsid (N), phosphoprotein (P), matrix, small hydrophobic (SH), surface attachment glycoprotein (G), surface fusion glycoprotein (F), an M2 gene which encodes two proteins from M2-1/M2-2 open reading frames, and an RNA-dependent RNA polymerase (L) [5,6]. RSV is surrounded by a lipid envelope derived from the host plasma membrane during viral budding [7]. The viral surface proteins, i.e., G, F, and SH, have been associated with modifying the host response to infection [8]. The G protein is a glycosylated membrane protein that has an extracellular ectodomain, containing a central conserved domain (CCD) with four cysteine residues that are highly conserved in all RSV isolates [9]. This central conserved domain (CCD) contains a CX3C chemokine motif (amino acids 182–186) that facilitates RSV attachment to susceptible cells bearing a CX3C chemokine receptor, CX3CR1 [10,11,12,13]. This domain is flanked by two hypervariable mucin-like domains [8]. The G protein is both membrane-bound (Gm) and soluble (Gs) [14]. An excess of the G protein is expressed as Gs which functions as an antigenic decoy, and this molecule retains the same characteristics as Gm based on glycosylation and antibody reactivity [15,16]. RSV attachment to cells in cell culture occurs via heparin-binding domains on the G protein with cell surface glycosaminoglycans, GAGs [17], although the G protein is not required for RSV attachment to cultured cells as shown by RSV mutant viruses lacking the G gene [18]. However, RSV attachment to airway epithelial cells in vivo occurs via G protein binding to CX3CR1, and RSV mutant viruses lacking G are highly attenduated in vivo [18,19]. The F protein is necessary for infection and replication, as RSV mutants lacking the F gene do not replicate [20]. RSV may infect a variety of cell types but primarily infects epithelial cells in the respiratory tract where infection results in cytokine and chemokine gene expression, pulmonary inflammation, and innate and adaptive immune defenses that regulate virus infection and replication [10,21,22,23,24,25].

Unfortunately, there is no licensed RSV vaccine, and the only FDA-approved treatment is prophylaxis using palivizumab—a monoclonal antibody (mAb) specific to the RSV F protein that may reduce disease severity and hospitalization [26,27]. However, there are several small and large molecules at late stage under investigation [28]. Studies suggest that large molecule therapy using anti-RSV G and F protein mAbs are desirable to ameliorate RSV disease [29,30,31]. Importantly, some mAbs that target RSV G protein neutralize RSV infection of human airway epithelial (HAE) cells and reduce RSV viral loads and disease in animal models [30,32,33,34,35]. In addition, anti-G protein mAbs reduce pulmonary inflammation, proinflammatory cytokines, and mucus production, and restore Th1/Th2 cytokine balance [25,36,37,38,39]. The anti-G protein mAbs have been shown to react to linear epitopes in the G protein CCD as determined by epitope mapping [40,41], and mAbs targeting the G protein CCD are protective in prophylactic and post-infection animal models [35,36,42]. Thus, RSV G protein vaccines are under investigation in preclinical studies. 

The crystal structures of the broadly neutralizing human mAbs, 3G12 and 3D3, bound to RSV G protein CCD, have permitted the determination of interactions outside the linear epitope, revealing a role for a G protein cysteines in stabilizing conformational epitopes and contributing to high-affinity antibody binding [43,44]. The RSV G protein is important in RSV infection, as the G protein CCD contains a CX3C chemokine motif which facilitates binding to CX3CR1 [13,45], and CX3CL1 mimicry has been shown to facilitate RSV infection and alter CX3CL1-mediated chemotaxis of human and mouse leukocytes [10,13,19]. Accumulating studies such as these show that the G protein CCD and CX3C motif are important when designing an RSV vaccine. For example, immunization of mice with RSV G protein or G protein polypeptides incorporating the CCD induces a protective antibody response [46,47,48], and the CX3C motif is important for inducing protective immune responses [49,50]. However, one worry when developing RSV G protein vaccines is the general poor immunogenicity of the glycoprotein. The heavily glycosylated mucin-like domains flanking the CCD are poor inducers of protective antibodies, as improved immunogenicity occurs in non-glycosylated G protein vaccinated mice [51]. Thus, rationally designed mutations using a reverse vaccinology approach to enhance immunogenicity have been proposed.

It has been shown that an insertion in the G protein CX3C motif (CX4C) reduces CX3CR1 binding and has a markedly reduced disease severity in vivo [46]. In this study, we investigated if RSV G protein immunogens designed to enhance immunogenicity and avoid vaccine-induced enhanced disease would mediate blocking Abs, preventing RSV G protein CX3C-CX3CR1 interaction in a mouse model. Of the panel of immunogens tested, two CCD mutants (177Q and 177R) were compared to RSV G protein or a CX4C mutant immunogen and mutant 177Q generated a robust serum antibody response that inhibited CX3C-CX3CR1 binding and generated strong anti-RSV IgG responses. The data also shows that RSV G protein immunogens with intact CX3C motifs, despite mutations proximal to this motif, induce an immunogenic and CX3C-CX3CR1 blocking antibody response and do not interfere with endogenous fractalkine CX3C binding. These findings show that structurally guided RSV vaccine candidates with mutations within the CCD are immunogenic.

## 2. Materials and Methods 

### 2.1. Mice

Specific-pathogen-free, 6–8-week-old female BALB/c mice (Jackson Labs, Bay Harbor, ME, USA) were used in all studies. Mice were housed in microisolator cages and were fed food and water *ad libitum*. Mice were weighed daily. All experiments were performed in accordance with the guidelines of the University of Georgia Institutional Animal Care and Use Committee (IACUC), with protocols approved by the University of Georgia IACUC (Approved: 5 May 2018; Approval code: A2018 04-018-A2).

### 2.2. Immunogens 

A synthetic gene encoding RSV strain A2 (RSV/A2) G protein amino acids 64 to 298 (UnitProtKB entry P03423) was cloned into pCF in-frame with an N-terminal TPA signal sequence and C-terminal tandem 6-histidine and Twin-Strep purification tags. Recombinant RSV G protein was produced by transient-transfection in CHO-S cells (Thermo Fisher Scientific, Waltham, MA, USA #R800-07) and secreted RSV G protein was affinity purified on a StrepTrap column (GE Healthcare Bio-Sciences, Uppsala, Sweden). Mutants were produced by Phusion site-directed mutagenesis and verified by Sanger sequencing. The CX4C mutant contained an insertion of an alanine within the CX3C motif, making it to ^182^CWAIAC^187^. Mutants S177Q and S177R contained serine177 substitution with glutamine or arginine, respectively. All proteins were concentrated and dialyzed into PBS. Proteins were flash-frozen in liquid nitrogen and stored at −80 °C until use. SDS-polyacrylamide gel electrophoresis was used to evaluate protein purity.

### 2.3. Virus

RSV strain A2 (RSV/A2) was propagated in Vero E6 cells (ATCC CRL-1586) as previously described [52]. Mice were anesthetized by intraperitoneal administration of Avertin (200 mg/kg) and intranasally challenged with 10^6^ PFU of RSV/A2 in 30 μL PBS (Hyclone, South Logan, UT, USA). 

### 2.4. Vaccination

6–8-week-old female BALB/c mice (Jackson Labs, Bar Harbor, ME, USA) were intramuscularly vaccinated with 10 μg of immunogen or PBS + 10 μg synthetic monophosphoryl lipid A (MPLA, Invivogen, San Diego, CA, USA) diluted in PBS. Mice (*n* = 10 per group) were primed on day 0 and boosted on days 21 and 60 with homogenous vaccination. A subset (*n* = 4–5) of mice from each group were euthanized prior to the challenge. On day 72 (12 days post-boost), mice were i.n. challenged with 10^6^ PFU RSV/A2 and subsequently euthanized at 7 days post-challenge. 

### 2.5. RSV ELISA

For indirect ELISAs, flat-bottom high-binding ELISA plates (Costar, Corning, NY, USA) were incubated with 10^6^ PFU/mL RSV/A2 diluted in PBS overnight at 4 °C. The plates were washed three times with 1x KPL Wash Buffer (SeraCare, Gaithersburg, MD, USA) and blocked overnight at 4 °C with Blotto (5% non-fat dry milk + 1% bovine serum albumin) (BSA, Sigma Aldrich, St. Louis, MO, USA) in PBS. The blocking solution was decanted, and sera diluted 1:40 in Blotto was incubated in triplicate for 1 h at 37 °C. The solution was decanted and the wells were washed three times and incubated with goat anti-mouse HRP conjugated secondary (ThermoFisher, Waltham, MA, USA), IgG1 or IgG2a (SouthernBiotech, Birmingham, AL, USA) for 1 h at 37 °C. Plates were washed in PBS, developed with 1-Step Ultra TMB Substrate (ThermoFisher, Waltham, MA, USA), and the reaction was stopped with Stop Solution (Invitrogen, Carlsbad, CA, USA). The plates were read at OD_450_ using an ELISA plate reader (BioTek, Winooski, VT, USA). Graphs are representative of three independent experiments. Bars represent the mean + SEM. Background was subtracted and compared to the adjuvant-only group. 

### 2.6. Anti-G Protein Antibody ELISA

To deplete anti-RSV F protein Abs, the pooled sera were subjected to an AminoLink Plus Resin RSV F protein column (ThermoFisher, Waltham, MA, USA) as described by the manufacturer. Briefly, RSV F protein was coupled to an AminoLink Plus Resin column and the column was washed. The F protein coupling efficiency was > 76% and within range of the manufacturer’s expected yield. To enrich the anti-G protein Abs, the serum was added to the F protein-conjugated column, and the antibody flow-through (i.e., anti-G protein Abs) was collected and quantified by ELISA using secondary goat anti-mouse IgG-HRP (ThermoFisher, Waltham, MA, USA). Graphs are representative of three independent experiments. Each bar represents the mean + SEM of technical triplicates from representative experiment. Background was subtracted and compared to adjuvant only group and tested by one-way ANOVA. 

### 2.7. Fractalkine (FKN) and G Protein CX3C-CX3CR1 Binding Assay

Human 293 cells (CRL-1573; ATCC) were maintained in 10% FBS + DMEM and CX3CR1.293 cells (Genscript, Piscataway, NJ, USA) were maintained in 10% FBS +1 μg/mL puromycin in DMEM at 37 °C/5% CO_2_. To determine CX3CR1 expression, 2 × 10^5^ HEK293 (293) or CX3CR1.293 cells were washed in FACS Buffer (1% BSA in PBS). Cells were blocked for 20 min with 1 μg/mL Fc Block (BD Biosciences, Franklin Lakes, NJ, USA) and stained with anti-human CX3CR1-Alexa647 (BioLegend, San Diego, CA, USA) on ice. The cells were washed with FACS buffer and analyzed on LSR-II (BD Biosciences, San Jose, CA, USA). A minimum of 20,000 events were collected per experiment. 

To determine binding to CX3CR1, 20 nM FKN-biotin (AcroBiosystems, Newark, DE, USA) or 500 nM RSV G protein purified as previously described were tested [13]. Briefly, RSV G protein was purified from RSV/A2 infected Vero E6 cell lysate as described [49]. Supernatant containing RSV G protein was filtered through Hi-Trap *N*-hydroxysuccinimide (NHS)-activated column (GE Healthcare, Chicago, IL, USA) coupled to anti-RSV G protein monoclonal antibody (131-2G). The column was equilibrated with PBS plus 0.2% *N*-octyl-β-glycoside by fast-performance liquid chromatography (FPLC) (GE Healthcare, Chicago, IL, USA). Fractions were collected and pooled, neutralized with 2 M Tris, pH 8.0, and dialyzed overnight at 4 °C against PBS, pH 7.4. No F protein was detectable by Western blot, and no detergent after dialysis. FKN-biotin or RSV G protein were pre-incubated ±5 μg/mL heparin (Sigma-Aldrich, St. Louis, MO, USA) for 1 h on ice. 2 × 10^5^ 293 or CX3CR1.293 were blocked for 20 min with 1 μg/mL Fc block on ice. Cells were resuspended in FKN-biotin or G protein ± heparin on ice. FKN was detected using Streptavidin-PE (BioLegend, San Diego, CA, USA). To detect RSV G protein, cells were incubated with 10 μg/mL of anti-RSV G protein mAb (clone 130-5F) on ice, the cells washed in FACS buffer, and resuspended in goat anti-mouse Alexa488 (ThermoFisher, Waltham, MA, USA). Following 30 min incubation, the cells were washed and resuspended in FACS buffer and analyzed on LSR-II. 20,000 events were collected and gated for singlets. 

### 2.8. Inhibition of FKN and G Protein CX3C-CX3CR1 Binding

IgG was purified from the pooled sera of pre-challenge vaccinated mice (*n* = 5) using Protein G DynaBeads (ThermoFisher, Waltham, MA, USA) as described [53] to normalize for the same concentrations of IgG, and to remove endogenous CX3CL1 and other serum factors which might affect RSV G protein binding to CX3CR1. IgG was quantified by Take3 Cassette (BioTek, Winooski, VT, USA). Briefly, 20 nM of biotinylated-FKN or 500 nM RSV G protein was co-incubated for 1 h at 4 °C with 500 ng of IgG from the pooled sera of vaccinated mice and ±5 μg/mL heparin (Sigma-Aldrich, St. Louis, MO, USA) was used to block non-specific binding. IgG from normal unvaccinated mouse sera and IgG mAb from clone 131-2G reactive to RSV G protein was used as negative or positive controls, respectively. 2 × 10^5^ 293 or CX3CR1.293 cells were washed in FACS buffer and blocked with 1 μg/mL Fc Block (BD Biosciences, San Jose, CA, USA) for 20 min at 4 °C. Cells were resuspended in purified IgG and RSV G protein or FKN ± heparin for 45 min at 4 °C. Cells were washed and resuspended in PE-Streptavidin (BioLegend, San Diego, CA, USA) to detect FKN. 10 μg/mL of mAb from clone 130-5F was used to bind RSV G protein for 45 min at 4 °C. Cells were washed in FACS buffer and goat anti-mouse Alexa Fluor-488 (ThermoFisher, Waltham, MA, USA) added to detect bound 130-5F mAb for 45 min at 4 °C. Cells were washed and resuspended in FACS buffer and 20,000 events were acquired on the LSR-II (BD Biosciences, San Jose, CA, USA) flow cytometer. The percent specific inhibition of binding to CX3CR1 was calculated by the formula:

[1 − (% Alexa Fluor-488^+^ CX3CR1.293 cells treated with FKN or G protein + antibody mixture)/(% Alexa Fluor-488^+^ CX3CR1.293 cells treated with FKN or G protein + normal mouse IgG)] × 100, as previously described [47].

### 2.9. Bronchoalveloar Lavage (BAL) and Flow Cytometry 

To determine the bronchoalveolar cell infiltrates in vaccinated mice at 7 days post-challenge, the lungs were washed three times with 1mL ice cold PBS. BAL was centrifuged at 500× *g* for 5 min at 4 °C. Supernatants were transferred to tubes and stored at −80 °C. BAL cells were washed and resuspended in FACS buffer and counted using hemocytometer (ThermoFisher, Waltham, MA, USA) and vialbility determed by trypan blue exclusion. BAL cells were analyzed for cell size and granularity using FSC/SSC as previously described [54]. 20,000 events were collected on LSR-II (BD Biosciences, San Jose, CA, USA) and data were analyzed using FlowJo 10.6 (Becton, Dickinson and Company, Ashland, OR, USA). 

### 2.10. Quantitiatve Real-Time Polymerase Chain Reaction (qRT-PCR) 

Lung homogenates from vaccinated and challenged mice were prepared from lungs collected in ice-cold HBSS, and dissociated with gentleMACS C Tubes (Miltenyi Biotec, Bergisch Gladbach, Germany) to obtain single-cell suspension and stored at −80 °C until assayed. RNA was extracted from lung homogenates by RNAzol RT (Sigma Aldrich, Darmstadt, Germany) as described by manufacturer. RNA was quantified by Qubit RNA Broad-Range Assay Kit (Thermo Fisher Scientific, Waltham, MA, USA), and cDNA was synthesized by LunaScript RT SuperMix Kit (New England Biolabs, Ipswich, MA, USA). Expression of RSV matrix (M) gene was determined using primers and probes for the M gene were as follows: forward primer, 5′-GGC AAA TAT GGA AAC ATA GCT GAA-3′; reverse primer, 5′-TCT TTT TCT AGG ACA TTG TAY TGA ACA G-3′; probe, 5′-6-carboxyfluorescein (FAM)-TGT CCG TCT TCT ACG CCC TCG TC-black hole quencher 1 (BHQ-1)-3′, and GoTaq qPCR Master Mix (Promega, Madison, WI, USA) with the following cycling conditions: 120 s × 95 °C, 15 s × 95 °C and 60 s × 60 °C for 40 cycles. Standard curve was obtained using serial dilutins of known plaque-forming units (PFU) of RSVA/2 RNA. Threshold cycles (Ct) values for each sample were converted to genome equivalents using the standard curve. Each PCR was run three independent times with technical duplicates. 

### 2.11. Statistics

All statistical analyses were performed using GraphPad software (San Diego, CA, USA). The data is presented as the mean ± standard error of the mean (SEM). *p*-values < 0.05 were considered statistically significant. Statistical tests are indicated in the figure legends.

## 3. Results

### 3.1. Rationally Designed RSV G Protein Immunogens 

**Figure 1:**
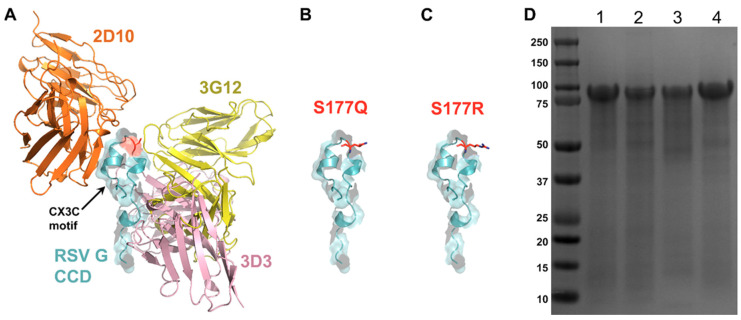
Rational design and expressiom of RSV G protein immunogens. (**A**) RSV G protein central conserved domain (CCD) (cyan) with CX3C motif highlighted, and sites of anti-G protein mAb binding: 2D10 (orange), 3G12 (yellow), and 3D3 (magenta). Serine 177 mutations modeled with (**B**) glutamine and (**C**) arginine. (**D**) Coomassie-stained SDS-PAGE gel of RSV G protein immunogens at ~90kDa. Lane 1: wild-type, lane 2: CX4C, lane 3: 177R, lane 4: 177Q.

RSV G protein contains glycosylated mucin-like regions which flank an unglycoslyated CCD (aa 157–198), and this domain has been shown to have low immunogenicity [55] which presents some difficulty when designing vaccine immunogens. Our previous structural analysis of the G protein when bound to neutralizing mAbs, 3D3 and 2D10 allowed us to map the antigenic regions (gamma 1 and gamma 2) to conformational epitopes [43,44]. Further, we showed that treatment with 3D3 and 2D10 mAbs could reduce RSV G protein CX3C-CX3CR1 chemotaxis by THP-1 monocytic cells after pre-treating with anti-G protein mAbs (3D3 and 2D10) [44]. In this study, a structure-guided immunogen approach was used to design RSV G protein mutants that are predicted to reduce CX3C interaction with CX3CR1 while increasing immunogenicity, and induce RSV G-CX3CR1 blocking antibodies. We selected serine 177 for mutagenesis, as this residue lies within the CCD but does not contribute to known conformational epitopes [43,45,56] (Figure 1A–C). We also generated the mutant CX4C, which had previously been shown to improve immunogenicity in the context of infection by RSV containing the CX4C mutation [46]. 

Recombinant RSV G protein immunogens were expressed in CHO-S cells and secreted G proteins were affinity purified from the media. This production method yielded G proteins of expected size (considering its heavy *N*- and *O*-glycosylation modifications) as determined by SDS-PAGE (Figure 1D). Immunogens were dialyzed into PBS and stored at −80 °C until vaccination.

### 3.2. Immunogencity of Immunogens 

**Figure 2:**
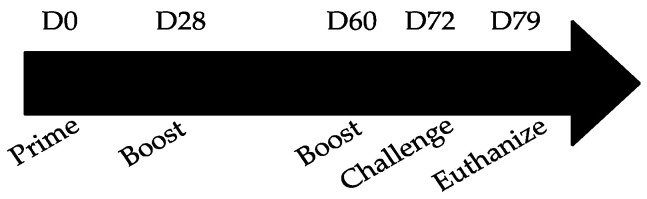
Outline of vaccination and challenge scheme. Mice were i.p. vaccinated with 10 μg/immunogen + 10 μg MPLA on days 0, 28, and 60. Mice were i.n. challenged (D72) with 10^6^ PFU RSV/A2 and sera were collected prior to and 7 days post-challenge (D79).

**Figure 3:**
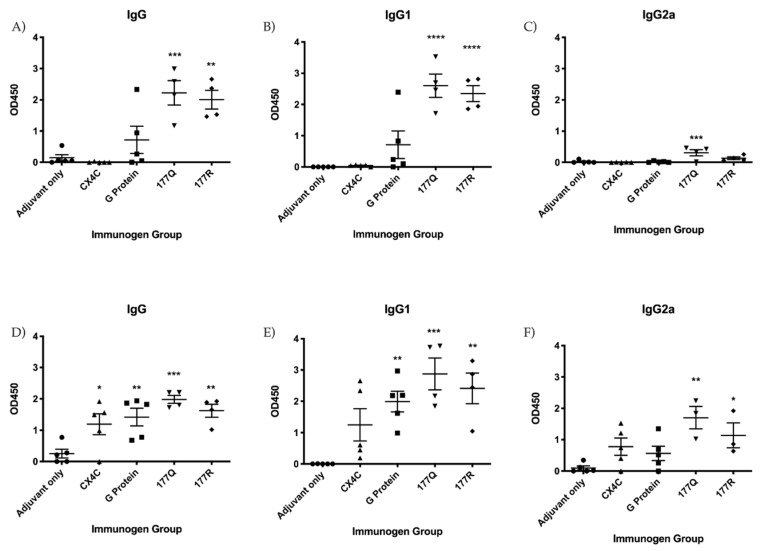
Immunogen vaccination induces anti-RSV IgG. Antibody levels were detected by indirect anti-RSV ELISA specific for total IgG (**A**,**D**); IgG1 (**B**,**E**); IgG2a (**C**,**F**) on days 0 (**A–C**) and day 7 (**D–F**) post-challenge. Graphs are representative of three independent experiments. Data represents the mean value of 3 experiments subtracted from the background. Bars represent the mean OD450 + SEM. *n*= 3–5 animals per group. Data were analyzed by one-way ANOVA tests where * *p* < 0.05, ** *p* < 0.01, *** *p* < 0.001, **** *p* < 0.0001 compared to adjuvant-only group.

To assess immunogenicity, mice were vaccinated using a prime-boost-boost scheme as outlined in Figure 2. Briefly, naïve BALB/c mice were intraperitoneally (i.p.) vaccinated with 10 μg MPLA (InvivoGen, San Diego, CA, USA) mixed with 10 μg of the individual immunogen (i.e., G protein, CX4C, 177Q, or 177R) or sham-treated (adjuvant only). MPLA is a TLR4-specific agonist which has shown to be promising in other RSV vaccine studies inducing a Th1-biased response [56]. Sera were collected 12 days after the second boost and assayed for anti-RSV and anti-RSV G protein Abs. Figure 3A shows the total IgG detected against RSV in the vaccinated sera. Interestingly, only 177Q or 177R immunogens induced a significant (177Q; *p* = 0.004; 177R *p* = 0.001) anti-RSV response. Figure 3B,C show IgG1 and IgG2a anti-RSV levels, respectively, and the robustness of antibody response. The G protein mutant candidates induced a significant IgG1 response (177Q, 177R *p* < 0.001), and 177Q had significantly (*p* = 0.008) higher IgG2a levels that correlated with a Th1-type response [57].

The IgG serum antibody response recalled after vaccination was evaluated seven days post-challenge (Figure 3D,F). G protein, CX4C, 177Q, and 177R immunogens significantly (G protein, *p* = 0.008; CX4C, *p* = 0.036; 177Q, *p* = 0.004; 177R: *p* = 0.003) induced higher antibody responses compared to adjuvant-only (Figure 3). It has been previously shown that the CX4C immunogen is immunogenic inducing a effective anti-RSV serum antibody response [47]. Interestingly, the IgG1 serum antibody responses were significantly increased by G protein (*p* = 0.006), 177Q (*p* = 0.0004) or 177R (*p* = 0.002) immmunogens, and the IgG2a antibody levels were significantly increased for the 177Q (*p* = 0.002) and 177R (*p* = 0.048) immunogens compared to adjuvant. These data show that despite mutations in the G protein, the immunogens were immunogenic and able to induce a clear humoral anti-RSV response.

**Figure 4:**
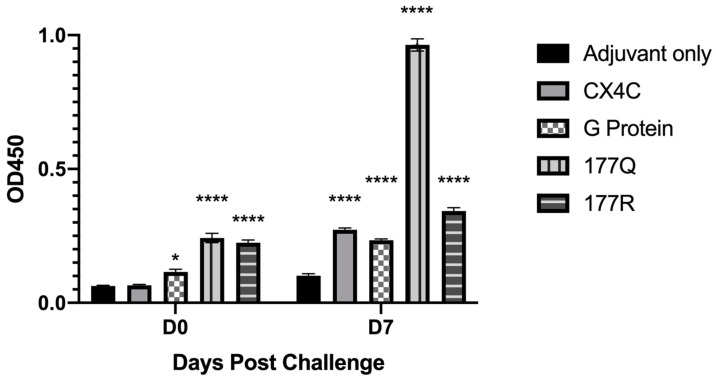
RSV G protein enriched sera. Antibody levels were detected by indirect anti-RSV IgG ELISA. Sera from *n* = 3–5 animals/group were panned against F protein to remove anti-F Abs prior to analysis by indirect ELISA. Bars represent the mean + SEM. Data were analyzed by one-way ANOVA tests where * *p* < 0.05, **** *p* < 0.0001 compared to adjuvant only group in three independent experiments.

To evaluate anti-G protein IgG responses in vaccinated mice, sera from the individual immunogen vaccinated mice was depleted of anti-F protein Abs. Figure 4 shows the total IgG detected by ELISA in the anti-F protein panned sera prior to and at seven days post-challenge. Consistent with the results in Figure 3A, the sera from 177Q and 177R vaccinated mice prior to the RSV challenge had significantly (*p* < 0.0001) higher anti-RSV Ab titers compared to sera from adjuvant-only immunized mice, and G protein reached significance (*p* = 0.0189). At 7 days post-challenge, all sera from immunogen vaccine groups was substantially higher than the adjuvant-only group which was consistent with the total IgG as shown in Figure 3D. These data show that the anti-RSV Ab response is increased in all immunogen groups that embody anti-G protein Ab responses. 

### 3.3. Ab Blocking of FKN-CX3CR1 and G Protein CX3C-CX3CR1 Interaction

**Figure 5:**
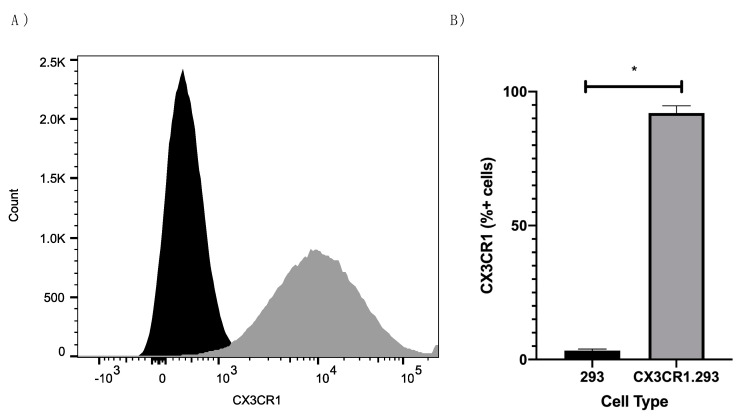
CX3CR1.293 cells abundantly express CX3CR1. Representative histogram (**A**) and percentages (**B**) of CX3CR1 expression in 293 (black) and CX3CR1.293 (gray) cells after staining with anti-CX3CR1-Alexa647. 20,000 events were collected. Bars represent the mean of three independent experiments + SEM where * *p* < 0.001 by two-tailed T test.

As the G protein immunogens induced robust anti-RSV and anti-RSV G protein Ab responses, we determined whether the Abs could inhibit RSV G protein CX3C-CX3CR1 binding and/or FKN (CX3CL1)-CX3CR1 binding. This is relevant because the G protein CX3C motif competes with FKN for binding to CX3CR1 [13] and affects antiviral CX3CR1^+^ cells trafficking to the site of infection [24]. Due to the lower CX3CR1 levels expressed by immortalized cells including 293 cells [11,13], 293 cells were stably transfected with CX3CR1 (CX3CR1.293) and were evaluated for their ability to bind G protein CX3C and FKN CX3C. Figure 5 shows that CX3CR1.293 cells express high levels of CX3CR1 (92%) compared to parent 293 cells (3%), thus we evaluated CX3C-CX3CR1 binding by RSV G protein and FKN and determined the binding inhibition mediated by IgG Abs induced by RSV G immunogens. 

**Figure 6:**
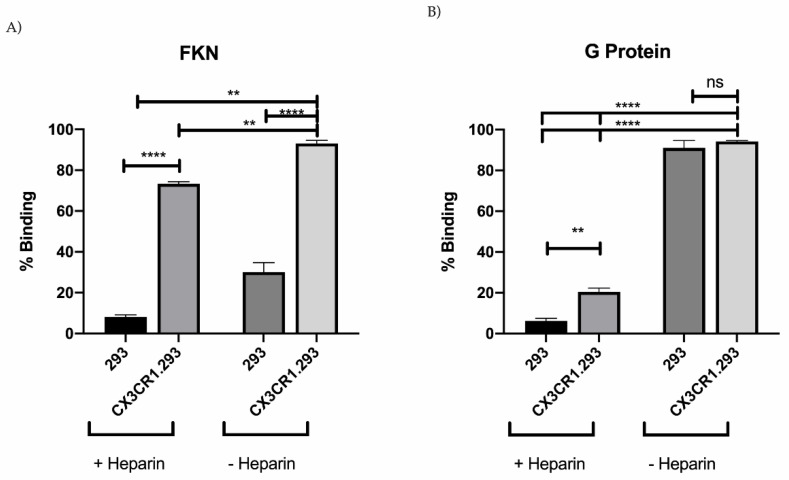
FKN and G protein CX3C binding to CX3CR1 in the presence or absence of heparin. 20 nM FKN (CX3CL1) (**A**) or 500 nM G protein (**B**) was pre-incubated with or without 5 μg/mL heparin to block non-specific binding. FKN binding was observed using Streptavidin-PE. RSV G protein was observed using anti-G protein mAb (clone 130-5F) followed by secondary anti-mouse conjugated to AlexaFluor-488. 20,000 events were collected. Bars represent the mean of three independent experiments + SEM analyzed by one-way ANOVA where ** *p* < 0.01, **** *p* < 0.0001, ns (no significance).

RSV G protein is glycosylated and contains heparin binding domains (HBD) which binds to cell cell surface gylcoaminoglycans (GAGs) [58]. Thus, blocking of non-specific G protein binding to the cells was done using 5 μg/mL heparin, which has been shown to aid the specific determination of RSV or G protein CX3C-CX3CR1 binding [11,13]. As expected, RSV G protein CX3C-CX3CR1 binding in the absence of heparin was affected by GAG interaction as binding was reduced in the presence of heparin (21%) yet was significantly (*p* = 0.0018) higher compared to 293 cell binding (6%) (Figure 6B). Importantly, FKN binding was dependent on CX3CR1 expression [59], and in the absence of heparin, FKN binding to CX3CR1.293 cells was 93% while 30% was bound non-specifically to 293 cells. In contrast, in the presence of heparin, FKN binding to CX3CR1 was reduced to 73% while binding to 293 cells was 8% (Figure 6A). These data show that FKN and RSV G protein bind to CX3CR1.293 cells with specificity. 

**Figure 7:**
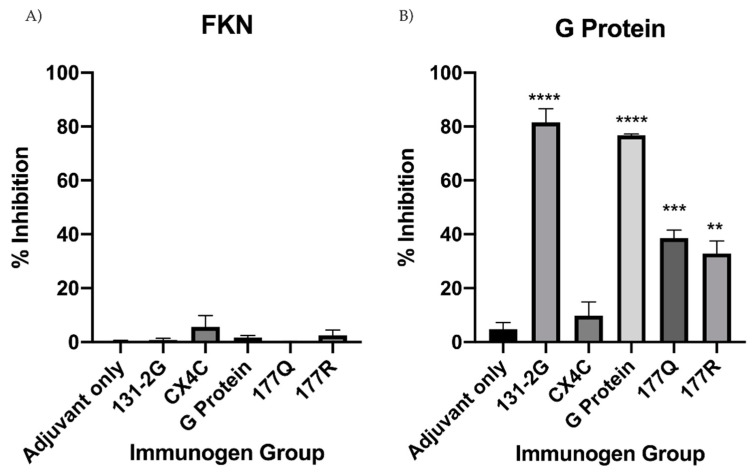
RSV G protein CX3C-CX3CR1 binding is inhibited by serum IgG from G protein immunogen vaccinated mice. Purified serum IgG from G protein immunogen or adjuvant vaccinated mice was examined for the ability to inhibit (**A**) FKN or (**B**) G protein CX3C binding to CX3CR1. IgG was co-incubated with FKN or G protein and 5 μg/mL heparin to block non-specific binding. Ligand-specific binding and percent inhibition was determined by: [1 − (% Alexa Fluor-488+ CX3CR1.293^+^ cells treated with ligand + antibody mixture) / (% Alexa Fluor-488+ CX3CR1.293 cells treated with ligand + normal mouse IgG)] × 100, as previously described. Bars represent mean of at least three independent experiments + SEM. Analysis by one-way ANOVA compared to adjuvant-only control where, ** *p* < 0.01, *** *p* < 0.001 **** *p* < 0.00001.

To determine if anti-RSV Abs could block CX3C-CX3CR1 interaction, IgG was purified from pooled immunogen vaccinated sera, quantified, and equal concentrations of IgG were used throughout the study. Then, 500 ng of purified IgG was incubated with either 20 nM FKN or 500 nM RSV G protein in the presence of 5 μg/mL heparin. The concentration chosen was determined from a gradient of FKN or G protein concentrations that were evaluated for CX3CR1.293 cell binding, which was consistent with related binding studies [49]. Binding of FKN or RSV G protein CX3C binding to CX3CR1 was determined by flow cytometery [47]. No measureable IgG tested inhibited FKN binding to CX3CR1 (Figure 7A). Importantly, IgG purified from G protein, 177Q, and 177R vaccinated mice significantly (G protein, *p* < 0.0001; 177Q, *p* = 0.0003; 177R, *p* = 0.0013) inhibited RSV G protein CX3C-CX3CR1 interaction compared to adjuvant-only (Figure 7B). Further, IgG from CX4C vaccinated sera was unable to substantially inhibit RSV G protein CX3C-CX3CR1 binding (*p* = 0.88) suggesting the need to retain the CX3C motif to induce blocking Abs as shown by the findings from G proten mutants, 177Q and 177R, which were able to induce blocking Abs. 131-2G is mAb known to bind to RSV G protein [60], and block RSV G protein CX3C-CX3CR1 interaction [13,61], and as expected did not interfere with FKN binding to CX3CR1 while significantly (*p* < 0.0001) inhibiting G protein CX3C binding to CX3CR1. These data show that the IgG from the sera of mice vaccinated with an intact CX3C motif induces blocking Abs that inhibit RSV G protein CX3C motif binding to CX3CR1.

### 3.4. G Protein Immunogen Safety and Enhanced RSV Disease

**Figure 8:**
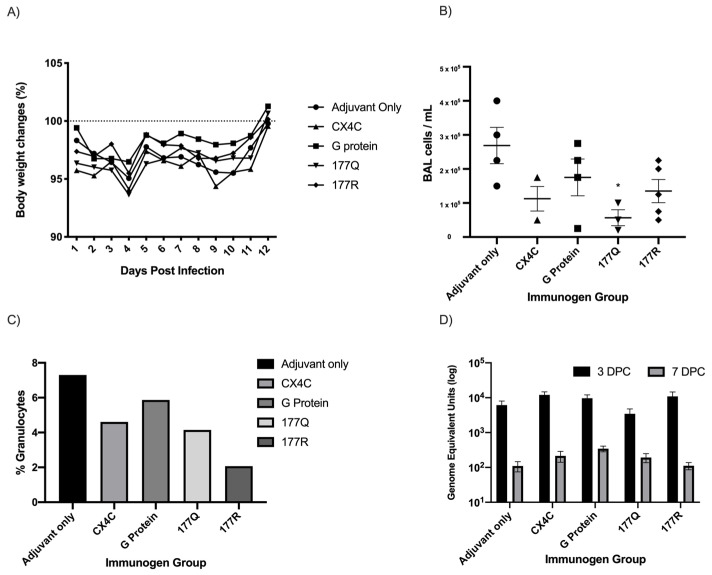
Disease markers in challenged mice. Mice were vaccinated with G protein immunogens and challenged with 10^6^ PFU RSV/A2. (**A**) Mice were weighed daily from days 0–12 post-challenge. Each shape represents the mean weight change from starting weight in each group each day. (**B**) Pulmonary leuokcyte infiltrates were collected seven days post-challenge. Graph indicates the mean BAL cells/mL + SEM. * *p* < 0.05 by one-way ANOVA compared to adjuvant-only group. (**C**) BAL cells were pooled and analyzed by flow cytometry. Percent granulocytes were determined using SSC^hi^ singlets. Twenty-thousand events were collected on LSR-II. (**D**) Lungs of challenged mice were collected on days 3 and 7 post-challenge and evaluated for RSV M protein transcripts by qRT-PCR. Data represent the mean of triplicate experiments. Standard curve was generated with a known concentration of RSV/A2 and two-way ANOVA was performed.

RSV G protein is known to modulate host immunity and cause pathogensis [12,24,25,62]. To determine if immunization with G protein immunogens enhanced RSV disease in mice, the weight, BAL cell infiltration, and pulmonary viral load were measured following RSV/A2 challenge. It is known that FI-RSV vaccine or Gs protein priming followed by RSV challege in mice leads to enhanced disease and weight loss [33,51,63,64,65], and a recent report suggests that the G protein CX3C motif enhances disease similar to that of of FI-RSV priming [66,67]. Thus, to address G protein vaccine safety, G protein immunogen vaccinated mice were weighed daily following RSV infection. None of the G protein immunogen or sham vaccinated mice showed substantial weight loss following RSV challenge, and all groups returned to near 100% weight by day 12 post infection (Figure 8A).

RSV lung pathology in mice is accompanied by a granulcytic BAL cell infiltrate e.g., eosinophils [68,69,70]. To evaluate the pulmonary cellular influx, BAL was collected from G protein immunogen vaccinated mice at seven days post-challenge (Figure 8B). Notably, BAL cells from 177Q vaccinated mice had significantly (*p* = 0.019) lower BAL leukocytes compared to adjuvant-only vaccinated mice, suggesting this vaccine does not prime for extensive pulmonary cellular responses when RSV-challenged. No other immunogen group had significantly (*p* > 0.05) reduced BAL cell influx when RSV-challenged. The BAL cells were collected andd pooled and analyzed by flow cytometry for cell size and granularity (forward vs side scatter) as described [54], and the number of granulocytes was determined by SSC^hi^ singlets (Figure 8C). The percentage of BAL granulocytes were highest in adjuvant only vaccinated mice (7.3%), followed by G protein (5.7%), CX4C (4.6%), 177Q (4.2%), and 177R (2.1%). These data suggest that the G protein immunogens induce a temperate response with minimal pulmonary leukocyte influx—a feature that may be linked to the antibody response (Figure 3 and Figure 4) with the ability to block CX3C-CX3CR1 interaction (Figure 7). To determine if vaccination reduces viral load, the lungs of infected mice were analyzed at three and seven days post-challenge and evaluated for RSV matrix (M) gene transcripts by real-time quantitative reverse transcription-PCR (qRT-PCR) as previously described [23,33,46]. Figure 8D shows no substantial differences in the pulmonary viral load in vaccinated groups on either days three or seven days post-challenge.

## 4. Discussion

Given the need for a safe and efficacious RSV vaccine, several studies are currently evaluating various vaccine candidates using different platforms, i.e., codon deoptimized live attenuated, subunit, particle, and vector-based, using different RSV antigens including F protein, G protein, and/or M protein [28,71,72,73]. While the F protein is the RSV vaccine antigen often evaluated, the G protein should also be considered as it can induce neutralizing activities and block CX3C-CX3CR1 interaction [42,47,48,74,75]. RSV vaccine development has been hampered by the difficulty in balancing vaccine immunogenicity and safety, primarily because of the early results in developing an FI-RSV vaccine that led to enhanced disease and some infant mortality [66,76,77]. Preclinical studies have shown RSV G protein can induce Abs that reduce the enhanced disease noted in FI-RSV vaccination, highlighting the potential of G protein as a vaccine candidate [78]. Another important factor in inducing anti-G protein antibodies in a vaccine is the potential to combat the solube G protein function as an immune modulator. An alternative translation site (Met48) results in secreted G protein, which is conserved between subtypes of the virus [8]. This early expressed soluble protein functions as an antigenic decoy, and reduces a protective host immune response by trafficking CX3CR1^+^ leukocytes [24,41]. There have also been failures in RSV vaccine development linked to poor immunogenicity and/or safety concerns [79]. Further complicating RSV vaccine development are the distinct vaccine target populations, i.e., infants, young children, elderly, and pregnant women, which may require different vaccines and/or approaches [80]. Thus, the path towards developing a safe and effective RSV vaccine is challenging. 

In the present study, RSV G protein immunogens were used in a prime/boost/boost scheme to vaccinate mice and determine their potential as a vaccine. Immunogens were designed using a structure-guided, reverse-vaccinology method using an in silico design [81,82,83]. The design was based on enhancing immunogenicity, inducing anti-G protein CX3C-CX3CR1 blocking Abs, and reducing the potential for FKN CX3C-CX3CR1 hindrance. Previous studies from our lab have suggested a reverse-vaccinology approach may be beneficial for vaccine development where previous technologies have failed [84]. Two structure-guided G protein mutants (177Q, 177R) having CX3C motifs but substitutions at 177aa (proximal to the CX3C motif) were tested to determine if anti-RSV G protein Ab responses induced by vaccination could block CX3C-CX3CR1. As a control, a CX4C mutant that ablated CX3C motif, but was otherwise identical to G protein CCD, was evaluated. Ab responses to 177Q and 177R showed retention of immunogenicity while having the ability to reduce CX3C-CX3CR1 interaction while G protein vaccination had lower responses. These results suggest that the mutations enhanced the immunogenicity of the region, and are consistent with the findings that G protein CCD is poorly immunogenic [41]. Further, 177Q had significantly increased IgG1 and respectable IgG2a responses compared to the adjuvant only group prior to challenge. 

As a means to evaluate the potential to block G protein CX3C-CX3CR1 interaction while not interfering with endogenous chemokine induced binding, a CX3CR1.293 cell line which expresses substantial CX3CR1 was developed. None of the IgG from the vaccinated mice were able to block FKN binding to CX3CR1 suggesting these antibodies will not interfere with FKN signaling in vivo. Importantly, all immunogens with intact CX3C motifs, despite the proximal mutations in mutants 177Q and 177R, induced an antibody response which significantly blocks G protein CX3C binding the CX3CR1. The blocking of this motif through both prophylactic/therapeutic mAbs and vaccines has shown promise by reducing CX3C-mediated disease in mice and preclinical trials [47,48,71,74,85]. To evaluate protection from enhanced disease, mice were challenged with RSV/A2 and disease parameters including weight loss, BAL cell inflitrate, and lung viral load were assessed. No significant weight loss post-challenge was observed in any vaccinated group, and all mice returned to baseline weight by 12 days post infection. Contrary to other studies noting enhanced weight loss with G protein priming followed by RSV challenge, we did not note any substantial weight loss perhaps mediated by the adjuvant or dosing of our immunogens. Also, there were no substantial differences in lung virus load isolated at three or seven days post-challenge. Importantly, 177Q vaccination resulted in reduced BAL cell infiltrates suggesting this vaccine, likely mediated by the substantial Ab response, may be able to reduce RSV disease. 

This study shows that using a structure-guided reverse vaccinology approach to generate mutations within the G protein may aid the development of RSV G protein vaccines with improved immunogenicity and safety. Despite demonstrating enhanced immunogenicity and CX3C-CX3CR1 blocking, there are limitations in this study which should be addressed. The female BALB/c mouse model is commonly used for immunogenicity studies, however this work cannot comment on any differences which may have been evident in male mice. There are also some limitations to inferring protection from disease using our present model. The BALB/c mouse is a semi-permissive model which at moderate inoculum does not present with robust disease [86]. A more investigative study into protection is warranted. Future studies should evaluate the in vivo reduction of disease by measuring cytokine and T-cell responses, airway, and lung pathology in vaccinated mice after challenge with a mouse-adapted pathogenic RSV strain (r19F-RSV/A2) as previously performed [37,42].

## 5. Conclusions

These results suggest that structure-guided G protein immunogens can be used as a platform to develop RSV vaccines that can induce CX3C-CX3CR1 blocking antibodies as a strategy to develop safe and effective vaccine candidates.

## Data Availability

The data supporting reported results are available upon request.

## References

[1] Chatzis O., Darbre S., Pasquier J., Meylan P., Manuel O., Aubert J.D., Beck-Popovic M., Masouridi-Levrat S., Ansari M., Kaiser L. (2018). Burden of severe RSV disease among immunocompromised children and adults: A 10 year retrospective study. BMC Infect. Dis..

[2] Schildgen O. (2009). The lack of protective immunity against RSV in the elderly. Epidemiol. Infect..

[3] Coultas J.A., Smyth R., Openshaw P.J. (2019). Respiratory syncytial virus (RSV): A scourge from infancy to old age. Thorax.

[4] Piedimonte G., Perez M.K. (2014). Respiratory syncytial virus infection and bronchiolitis. Pediatr. Rev..

[5] Borchers A.T., Chang C., Gershwin M.E., Gershwin L.J. (2013). Respiratory syncytial virus—A comprehensive review. Clin. Rev. Allergy Immunol..

[6] Boyoglu-Barnum S., Chirkova T., Anderson L.J. (2019). Biology of Infection and Disease Pathogenesis to Guide RSV Vaccine Development. Front. Immunol..

[7] Collins P.L., Melero J.A. (2011). Progress in understanding and controlling respiratory syncytial virus: Still crazy after all these years. Virus Res..

[8] McLellan J.S., Ray W.C., Peeples M.E. (2013). Structure and function of respiratory syncytial virus surface glycoproteins. Curr. Top. Microbiol. Immunol..

[9] Kauvar L.M., Harcourt J.L., Haynes L.M., Tripp R.A. (2010). Therapeutic targeting of respiratory syncytial virus G-protein. Immunotherapy.

[10] Zhivaki D., Lemoine S., Lim A., Morva A., Vidalain P.O., Schandene L., Casartelli N., Rameix-Welti M.A., Herve P.L., Deriaud E. (2017). Respiratory Syncytial Virus Infects Regulatory B Cells in Human Neonates via Chemokine Receptor CX3CR1 and Promotes Lung Disease Severity. Immunity.

[11] Chirkova T., Lin S., Oomens A.G.P., Gaston K.A., Boyoglu-Barnum S., Meng J., Stobart C.C., Cotton C.U., Hartert T.V., Moore M.L. (2015). CX3CR1 is an important surface molecule for respiratory syncytial virus infection in human airway epithelial cells. J. Gen. Virol..

[12] Tripp R.A., Dakhama A., Jones L.P., Barskey A., Gelfand E.W., Anderson L.J. (2003). The G glycoprotein of respiratory syncytial virus depresses respiratory rates through the CX3C motif and substance P. J. Virol..

[13] Tripp R.A., Jones L.P., Haynes L.M., Zheng H., Murphy P.M., Anderson L.J. (2001). CX3C chemokine mimicry by respiratory syncytial virus G glycoprotein. Nat. Immunol..

[14] Hendricks D.A., Baradaran K., McIntosh K., Patterson J.L. (1987). Appearance of a soluble form of the G protein of respiratory syncytial virus in fluids of infected cells. J. Gen. Virol..

[15] Hendricks D.A., McIntosh K., Patterson J.L. (1988). Further characterization of the soluble form of the G glycoprotein of respiratory syncytial virus. J. Virol..

[16] Bukreyev A., Yang L., Collins P.L. (2012). The secreted G protein of human respiratory syncytial virus antagonizes antibody-mediated restriction of replication involving macrophages and complement. J. Virol..

[17] Hallak L.K., Spillmann D., Collins P.L., Peeples M.E. (2000). Glycosaminoglycan sulfation requirements for respiratory syncytial virus infection. J. Virol..

[18] Karron R.A., Buonagurio D.A., Georgiu A.F., Whitehead S.S., Adamus J.E., Clements-Mann M.L., Harris D.O., Randolph V.B., Udem S.A., Murphy B.R. (1997). Respiratory syncytial virus (RSV) SH and G proteins are not essential for viral replication in vitro: Clinical evaluation and molecular characterization of a cold-passaged, attenuated RSV subgroup B mutant. Proc. Natl. Acad. Sci. USA.

[19] Johnson S.M., McNally B.A., Ioannidis I., Flano E., Teng M.N., Oomens A.G., Walsh E.E., Peeples M.E. (2015). Respiratory Syncytial Virus Uses CX3CR1 as a Receptor on Primary Human Airway Epithelial Cultures. PLoS Pathog..

[20] Gonzalez-Reyes L., Ruiz-Arguello M.B., Garcia-Barreno B., Calder L., Lopez J.A., Albar J.P., Skehel J.J., Wiley D.C., Melero J.A. (2001). Cleavage of the human respiratory syncytial virus fusion protein at two distinct sites is required for activation of membrane fusion. Proc. Natl. Acad. Sci. USA.

[21] Tripp R.A. (2013). Respiratory Syncytial Virus (RSV) Modulation at the Virus-Host Interface Affects Immune Outcome and Disease Pathogenesis. Immune Netw..

[22] Schmidt M.E., Varga S.M. (2017). Modulation of the host immune response by respiratory syncytial virus proteins. J. Microbiol..

[23] Bakre A.A., Harcourt J.L., Haynes L.M., Anderson L.J., Tripp R.A. (2017). The Central Conserved Region (CCR) of Respiratory Syncytial Virus (RSV) G Protein Modulates Host miRNA Expression and Alters the Cellular Response to Infection. Vaccines.

[24] Harcourt J., Alvarez R., Jones L.P., Henderson C., Anderson L.J., Tripp R.A. (2006). Respiratory syncytial virus G protein and G protein CX3C motif adversely affect CX3CR1+ T cell responses. J. Immunol..

[25] Chirkova T., Boyoglu-Barnum S., Gaston K.A., Malik F.M., Trau S.P., Oomens A.G., Anderson L.J. (2013). Respiratory syncytial virus G protein CX3C motif impairs human airway epithelial and immune cell responses. J. Virol..

[26] Resch B. (2017). Product review on the monoclonal antibody palivizumab for prevention of respiratory syncytial virus infection. Hum. Vaccines Immunother..

[27] (1998). Palivizumab, a Humanized Respiratory Syncytial Virus Monoclonal Antibody, Reduces Hospitalization from Respiratory Syncytial Virus Infection in High-Risk Infants. Pediatrics.

[28] Bergeron H.C., Tripp R.A. (2020). Emerging small and large molecule therapeutics for respiratory syncytial virus. Expert Opin. Investig. Drugs.

[29] Fuentes S., Hahn M., Chilcote K., Chemaly R.F., Shah D.P., Ye X., Avadhanula V., Piedra P.A., Golding H., Khurana S. (2020). Antigenic Fingerprinting of Respiratory Syncytial Virus (RSV)-A-Infected Hematopoietic Cell Transplant Recipients Reveals Importance of Mucosal Anti-RSV G Antibodies in Control of RSV Infection in Humans. J. Infect. Dis..

[30] Haynes L.M., Caidi H., Radu G.U., Miao C., Harcourt J.L., Tripp R.A., Anderson L.J. (2009). Therapeutic monoclonal antibody treatment targeting respiratory syncytial virus (RSV) G protein mediates viral clearance and reduces the pathogenesis of RSV infection in BALB/c mice. J. Infect. Dis..

[31] Taylor G., Stott E.J., Bew M., Fernie B.F., Cote P.J., Collins A.P., Hughes M., Jebbett J. (1984). Monoclonal antibodies protect against respiratory syncytial virus infection in mice. Immunology.

[32] Boyoglu-Barnum S., Gaston K.A., Todd S.O., Boyoglu C., Chirkova T., Barnum T.R., Jorquera P., Haynes L.M., Tripp R.A., Moore M.L. (2013). A respiratory syncytial virus (RSV) anti-G protein F(ab′)2 monoclonal antibody suppresses mucous production and breathing effort in RSV rA2-line19F-infected BALB/c mice. J. Virol..

[33] Radu G.U., Caidi H., Miao C., Tripp R.A., Anderson L.J., Haynes L.M. (2010). Prophylactic treatment with a G glycoprotein monoclonal antibody reduces pulmonary inflammation in respiratory syncytial virus (RSV)-challenged naive and formalin-inactivated RSV-immunized BALB/c mice. J. Virol..

[34] Lee H.J., Lee J.Y., Park M.H., Kim J.Y., Chang J. (2017). Monoclonal Antibody against G Glycoprotein Increases Respiratory Syncytial Virus Clearance In Vivo and Prevents Vaccine-Enhanced Diseases. PLoS ONE.

[35] Caidi H., Harcourt J.L., Tripp R.A., Anderson L.J., Haynes L.M. (2012). Combination therapy using monoclonal antibodies against respiratory syncytial virus (RSV) G glycoprotein protects from RSV disease in BALB/c mice. PLoS ONE.

[36] Caidi H., Miao C., Thornburg N.J., Tripp R.A., Anderson L.J., Haynes L.M. (2018). Anti-respiratory syncytial virus (RSV) G monoclonal antibodies reduce lung inflammation and viral lung titers when delivered therapeutically in a BALB/c mouse model. Antivir. Res..

[37] Boyoglu-Barnum S., Chirkova T., Todd S.O., Barnum T.R., Gaston K.A., Jorquera P., Haynes L.M., Tripp R.A., Moore M.L., Anderson L.J. (2014). Prophylaxis with a respiratory syncytial virus (RSV) anti-G protein monoclonal antibody shifts the adaptive immune response to RSV rA2-line19F infection from Th2 to Th1 in BALB/c mice. J. Virol..

[38] Miao C., Radu G.U., Caidi H., Tripp R.A., Anderson L.J., Haynes L.M. (2009). Treatment with respiratory syncytial virus G glycoprotein monoclonal antibody or F(ab′)2 components mediates reduced pulmonary inflammation in mice. J. Gen. Virol..

[39] Han J., Takeda K., Wang M., Zeng W., Jia Y., Shiraishi Y., Okamoto M., Dakhama A., Gelfand E.W. (2014). Effects of anti-g and anti-f antibodies on airway function after respiratory syncytial virus infection. Am. J. Respir. Cell Mol. Biol..

[40] Cortjens B., Yasuda E., Yu X., Wagner K., Claassen Y.B., Bakker A.Q., van Woensel J.B.M., Beaumont T. (2017). Broadly Reactive Anti-Respiratory Syncytial Virus G Antibodies from Exposed Individuals Effectively Inhibit Infection of Primary Airway Epithelial Cells. J. Virol..

[41] Collarini E.J., Lee F.E., Foord O., Park M., Sperinde G., Wu H., Harriman W.D., Carroll S.F., Ellsworth S.L., Anderson L.J. (2009). Potent high-affinity antibodies for treatment and prophylaxis of respiratory syncytial virus derived from B cells of infected patients. J. Immunol..

[42] Boyoglu-Barnum S., Todd S.O., Chirkova T., Barnum T.R., Gaston K.A., Haynes L.M., Tripp R.A., Moore M.L., Anderson L.J. (2015). An anti-G protein monoclonal antibody treats RSV disease more effectively than an anti-F monoclonal antibody in BALB/c mice. Virology.

[43] Fedechkin S.O., George N.L., Castrejon A.M.N., Dillen J.R., Kauvar L.M., DuBois R.M. (2020). Conformational Flexibility in Respiratory Syncytial Virus G Neutralizing Epitopes. J. Virol..

[44] Fedechkin S.O., George N.L., Wolff J.T., Kauvar L.M., DuBois R.M. (2018). Structures of respiratory syncytial virus G antigen bound to broadly neutralizing antibodies. Sci. Immunol..

[45] Anderson C.S., Chu C.Y., Wang Q., Mereness J.A., Ren Y., Donlon K., Bhattacharya S., Misra R.S., Walsh E.E., Pryhuber G.S. (2020). CX3CR1 as a respiratory syncytial virus receptor in pediatric human lung. Pediatr. Res..

[46] Boyoglu-Barnum S., Todd S.O., Meng J., Barnum T.R., Chirkova T., Haynes L.M., Jadhao S.J., Tripp R.A., Oomens A.G., Moore M.L. (2017). Mutating the CX3C Motif in the G Protein Should Make a Live Respiratory Syncytial Virus Vaccine Safer and More Effective. J. Virol..

[47] Jorquera P.A., Oakley K.E., Powell T.J., Palath N., Boyd J.G., Tripp R.A. (2015). Layer-By-Layer Nanoparticle Vaccines Carrying the G Protein CX3C Motif Protect against RSV Infection and Disease. Vaccines.

[48] Jorquera P.A., Choi Y., Oakley K.E., Powell T.J., Boyd J.G., Palath N., Haynes L.M., Anderson L.J., Tripp R.A. (2013). Nanoparticle vaccines encompassing the respiratory syncytial virus (RSV) G protein CX3C chemokine motif induce robust immunity protecting from challenge and disease. PLoS ONE.

[49] Zhang W., Choi Y., Haynes L.M., Harcourt J.L., Anderson L.J., Jones L.P., Tripp R.A. (2010). Vaccination to induce antibodies blocking the CX3C-CX3CR1 interaction of respiratory syncytial virus G protein reduces pulmonary inflammation and virus replication in mice. J. Virol..

[50] Harcourt J.L., Karron R.A., Tripp R.A. (2004). Anti-G protein antibody responses to respiratory syncytial virus infection or vaccination are associated with inhibition of G protein CX3C-CX3CR1 binding and leukocyte chemotaxis. J. Infect. Dis..

[51] Fuentes S., Coyle E.M., Golding H., Khurana S. (2015). Nonglycosylated G-Protein Vaccine Protects against Homologous and Heterologous Respiratory Syncytial Virus (RSV) Challenge, while Glycosylated G Enhances RSV Lung Pathology and Cytokine Levels. J. Virol..

[52] Tripp R.A., Moore D., Jones L., Sullender W., Winter J., Anderson L.J. (1999). Respiratory syncytial virus G and/or SH protein alters Th1 cytokines, natural killer cells, and neutrophils responding to pulmonary infection in BALB/c mice. J. Virol..

[53] Bearden C.M., Book B.K., Sidner R.A., Pescovitz M.D. (2005). Removal of therapeutic anti-lymphocyte antibodies from human sera prior to anti-human leukocyte antibody testing. J. Immunol. Methods.

[54] Waris M.E., Tsou C., Erdman D.D., Zaki S.R., Anderson L.J. (1996). Respiratory synctial virus infection in BALB/c mice previously immunized with formalin-inactivated virus induces enhanced pulmonary inflammatory response with a predominant Th2-like cytokine pattern. J. Virol..

[55] Jones H.G., Ritschel T., Pascual G., Brakenhoff J.P.J., Keogh E., Furmanova-Hollenstein P., Lanckacker E., Wadia J.S., Gilman M.S.A., Williamson R.A. (2018). Structural basis for recognition of the central conserved region of RSV G by neutralizing human antibodies. PLoS Pathog..

[56] Lambert S.L., Aslam S., Stillman E., MacPhail M., Nelson C., Ro B., Sweetwood R., Lei Y.M., Woo J.C., Tang R.S. (2015). A novel respiratory syncytial virus (RSV) F subunit vaccine adjuvanted with GLA-SE elicits robust protective TH1-type humoral and cellular immunity in rodent models. PLoS ONE.

[57] Firacative C., Gressler A.E., Schubert K., Schulze B., Muller U., Brombacher F., von Bergen M., Alber G. (2018). Identification of T helper (Th)1- and Th2-associated antigens of Cryptococcus neoformans in a murine model of pulmonary infection. Sci. Rep..

[58] Feldman S.A., Hendry R.M., Beeler J.A. (1999). Identification of a linear heparin binding domain for human respiratory syncytial virus attachment glycoprotein G. J. Virol..

[59] Hatakeyama M., Imaizumi T., Tamo W., Yamashita K., Yoshida H., Fukuda I., Satoh K. (2004). Heparin inhibits IFN-gamma-induced fractalkine/CX3CL1 expression in human endothelial cells. Inflammation.

[60] Anderson L.J., Bingham P., Hierholzer J.C. (1988). Neutralization of respiratory syncytial virus by individual and mixtures of F and G protein monoclonal antibodies. J. Virol..

[61] Sullender W. (1995). Antigenic analysis of chimeric and truncated G proteins of respiratory syncytial virus. Virology.

[62] Tripp R.A., Power U.F., Openshaw P.J.M., Kauvar L.M. (2018). Respiratory Syncytial Virus: Targeting the G Protein Provides a New Approach for an Old Problem. J. Virol..

[63] Johnson T.R., Johnson J.E., Roberts S.R., Wertz G.W., Parker R.A., Graham B.S. (1998). Priming with secreted glycoprotein G of respiratory syncytial virus (RSV) augments interleukin-5 production and tissue eosinophilia after RSV challenge. J. Virol..

[64] Shingai M., Azuma M., Ebihara T., Sasai M., Funami K., Ayata M., Ogura H., Tsutsumi H., Matsumoto M., Seya T. (2008). Soluble G protein of respiratory syncytial virus inhibits Toll-like receptor 3/4-mediated IFN-beta induction. Int. Immunol..

[65] Jia R., Lu L., Liang X., Sun Z., Tan L., Xu M., Su L., Xu J. (2017). Poly(U) and CpG ameliorate the unbalanced T cell immunity and pneumonia of mice with RSV vaccine-enhanced disease. Biosci. Trends.

[66] Haynes L.M., Jones L.P., Barskey A., Anderson L.J., Tripp R.A. (2003). Enhanced disease and pulmonary eosinophilia associated with formalin-inactivated respiratory syncytial virus vaccination are linked to G glycoprotein CX3C-CX3CR1 interaction and expression of substance P. J. Virol..

[67] Openshaw P.J., Culley F.J., Olszewska W. (2001). Immunopathogenesis of vaccine-enhanced RSV disease. Vaccine.

[68] Su Y.C., Townsend D., Herrero L.J., Zaid A., Rolph M.S., Gahan M.E., Nelson M.A., Rudd P.A., Matthaei K.I., Foster P.S. (2015). Dual proinflammatory and antiviral properties of pulmonary eosinophils in respiratory syncytial virus vaccine-enhanced disease. J. Virol..

[69] Oshansky C.M., Zhang W., Moore E., Tripp R.A. (2009). The host response and molecular pathogenesis associated with respiratory syncytial virus infection. Future Microbiol..

[70] Castilow E.M., Olson M.R., Varga S.M. (2007). Understanding respiratory syncytial virus (RSV) vaccine-enhanced disease. Immunol. Res..

[71] Hu M., Bogoyevitch M.A., Jans D.A. (2020). Impact of Respiratory Syncytial Virus Infection on Host Functions: Implications for Antiviral Strategies. Physiol. Rev..

[72] Biagi C., Dondi A., Scarpini S., Rocca A., Vandini S., Poletti G., Lanari M. (2020). Current State and Challenges in Developing Respiratory Syncytial Virus Vaccines. Vaccines.

[73] Boyoglu-Barnum S., Tripp R.A. (2020). Up-to-date role of biologics in the management of respiratory syncytial virus. Expert Opin. Biol. Ther..

[74] Choi Y., Mason C.S., Jones L.P., Crabtree J., Jorquera P.A., Tripp R.A. (2012). Antibodies to the central conserved region of respiratory syncytial virus (RSV) G protein block RSV G protein CX3C-CX3CR1 binding and cross-neutralize RSV A and B strains. Viral Immunol..

[75] Goetsch L., Plotnicky-Gilquin H., Aubry J.P., De-Lys P., Haeuw J.F., Bonnefoy J.Y., Nguyen N.T., Corvaia N., Velin D. (2001). BBG2Na an RSV subunit vaccine candidate intramuscularly injected to human confers protection against viral challenge after nasal immunization in mice. Vaccine.

[76] Fulginiti V.A., Eller J.J., Sieber O.F., Joyner J.W., Minamitani M., Meiklejohn G. (1969). Respiratory virus immunization. I. A field trial of two inactivated respiratory virus vaccines; an aqueous trivalent parainfluenza virus vaccine and an alum-precipitated respiratory syncytial virus vaccine. Am. J. Epidemiol..

[77] Kim H.W., Canchola J.G., Brandt C.D., Pyles G., Chanock R.M., Jensen K., Parrott R.H. (1969). Respiratory syncytial virus disease in infants despite prior administration of antigenic inactivated vaccine. Am. J. Epidemiol..

[78] Rey G.U., Miao C., Caidi H., Trivedi S.U., Harcourt J.L., Tripp R.A., Anderson L.J., Haynes L.M. (2013). Decrease in formalin-inactivated respiratory syncytial virus (FI-RSV) enhanced disease with RSV G glycoprotein peptide immunization in BALB/c mice. PLoS ONE.

[79] Higgins D., Trujillo C., Keech C. (2016). Advances in RSV vaccine research and development—A global agenda. Vaccine.

[80] Anderson L.J., Dormitzer P.R., Nokes D.J., Rappuoli R., Roca A., Graham B.S. (2013). Strategic priorities for respiratory syncytial virus (RSV) vaccine development. Vaccine.

[81] Kwong P.D., DeKosky B.J., Ulmer J.B. (2020). Antibody-guided structure-based vaccines. Semin. Immunol..

[82] Heinson A.I., Woelk C.H., Newell M.L. (2015). The promise of reverse vaccinology. Int. Health.

[83] Masignani V., Rappuoli R., Pizza M. (2002). Reverse vaccinology: A genome-based approach for vaccine development. Expert Opin. Biol. Ther..

[84] Rappuoli R., Bottomley M.J., D’Oro U., Finco O., De Gregorio E. (2016). Reverse vaccinology 2.0: Human immunology instructs vaccine antigen design. J. Exp. Med..

[85] Liang B., Kabatova B., Kabat J., Dorward D.W., Liu X., Surman S., Liu X., Moseman A.P., Buchholz U.J., Collins P.L. (2019). Effects of Alterations to the CX3C Motif and Secreted Form of Human Respiratory Syncytial Virus (RSV) G Protein on Immune Responses to a Parainfluenza Virus Vector Expressing the RSV G Protein. J. Virol..

[86] Kong X., Hellermann G.R., Patton G., Kumar M., Behera A., Randall T.S., Zhang J., Lockey R.F., Mohapatra S.S. (2005). An immunocompromised BALB/c mouse model for respiratory syncytial virus infection. Virol. J..

